# Development, validation, and visualization of a web‐based nomogram for predicting the incidence of leiomyosarcoma patients with distant metastasis

**DOI:** 10.1002/cnr2.1594

**Published:** 2021-12-03

**Authors:** Zhehong Li, Junqiang Wei, Haiying Cao, Mingze Song, Yafang Zhang, Yu Jin

**Affiliations:** ^1^ Department of Traumatology and Orthopaedics Affiliated Hospital of Chengde Medical College Chengde China

**Keywords:** distant metastasis, leiomyosarcoma, overall survival, SEER program, web‐based nomogram

## Abstract

**Background:**

Leiomyosarcoma (LMS) is one of the most common soft tissue sarcomas. LMS is prone to distant metastasis (DM), and patients with DM have a poor prognosis.

**Aim:**

In this study, we investigated the risk factors of DM in LMS patients and the prognostic factors of LMS patients with DM.

**Methods and results:**

LMS patients diagnosed between 2010 and 2016 were extracted from the Surveillance, Epidemiology, and End Result (SEER) database. Patients were randomly divided into the training set and validation set. Univariate and multivariate logistic regression analyses were performed, and a nomogram was established. The area under the curve (AUC), calibration curve, and decision curve analysis (DCA) were used to evaluate the nomogram. Based on the nomogram, a web‐based nomogram is established. The univariate and multivariate Cox regression analyses were used to assess the prognostic risk factors of LMS patients with DM. Eventually, 2184 patients diagnosed with LMS were enrolled, randomly divided into the training set (n = 1532, 70.14%) and validation set (n = 652, 29.86%). Race, primary site, grade, T stage, and tumor size were correlated with DM incidence in LMS patients. The AUC of the nomogram is 0.715 in training and 0.713 in the validation set. The calibration curve and DCA results showed that the nomogram performed well in predicting the DM risk. A web‐based nomogram was established to predict DM's risk in LMS patients (https://wenn23.shinyapps.io/riskoflmsdm/). Epithelioid LMS, in uterus, older age, giant tumor, multiple organ metastasis, without surgery, and chemotherapy had a poor prognosis.

**Conclusions:**

The established web‐based nomogram (https://wenn23.shinyapps.io/riskoflmsdm/) is an accurate and personalized tool to predict the risks of LMS developing DM. Advanced age, larger tumor, multiple organ metastasis, epithelioid type, uterine LMS, no surgery, and no chemotherapy were associated with poor prognosis in LMS patients with DM.

## 1 INTRODUCTION

Leiomyosarcoma (LMS) is one of the most common soft tissue sarcomas, accounting for 12% of all soft tissue sarcomas.^1^ It is reported that LMS mainly occurs in 50–60 years old patients and is often involved in the uterus, retroperitoneal space, and soft tissue.^2^ About 25% of LMS patients will occur DM even through radical resection.^3^, ^4^ Okamoto et al. have reported that the common metastatic sites of LMS include lung, liver, and bone.^5^ Further studies have shown that lung is the most common metastatic site.^3^, ^5^ Some studies have demonstrated that the survival rate of LMS patients is improving after receiving chemotherapy and surgery. However, the prognosis of LMS patients with DM is still poor, and the 5‐year survival rate was less than 20%.^6^, ^7^, ^8^ Leiomyosarcoma accounts for about 0.12% of all tumors, and because of the low incidence rate of LMS, the risk factors, and prognostic factors of DM are not yet clear.^1^ Therefore, identifying high‐risk LMS patients who are at risk of developing DM is meaningful. Many studies have shown that malignant tumor prognosis can be more accurate, more effective, and more beneficial by using nomograms.^9^, ^10^ Takehara et al. have analyzed the clinical status and prognosis of uterine LMS patients.^6^ Xue et al. have investigated the prognosis of extremities LMS patients and established a prognostic nomogram.^11^ However, as far as we know, there is no research on building a web‐based nomogram to estimate the DM risk in LMS patients. Besides that, there are no studies to predict the prognosis of LMS with DM. Therefore, we intend to use the Surveillance, Epidemiology, and End Results (SEER) database to evaluate DM incidence and risk factors in LMS and establish a visualized web‐based nomogram.^12^ Furthermore, we intend to predict the prognostic factors of LMS patients with DM.

## 2 MATERIALS AND METHODS

### 2.1 Patients

The data included in the present study were downloaded from the SEER*Stat software version 8.3.6.^12^ Using the International Classification of Diseases for Oncology, 3rd edition (ICD‐O‐3), we identified all LMS patients (ICD‐O‐3 histologic type: 8890, 8891, 8893, 8896). We collected case diagnosis time between 2010 and 2016. The inclusive criteria were as follows: (1) patients with pathological diagnosis of LMS, (2) patients from the time of 2010–2016, according to the term “year of diagnosis,” (3) complete follow‐up information, no data loss. The exclusion criteria were as follows: (1) patients missing essential details, including grade, stage, tumor size, surgery, radiotherapy, chemotherapy, survival time, and marital status. (2) follow‐up status is missing. According to the ethics guidelines, neither informed consent nor approval of the ethics committee is required because we use public and anonymous data.

### 2.2 Data element

The following demographic and clinical characteristics were included: age, sex (Female and Male), race [white, black, and others (American Indian/AK Native, Asian/Pacific Islander)], marital status (married and unmarried), grade (I–II or III–IV), T stage (I–II or III–IV), N stage (N0 or N1), surgical treatment (No or Yes), radiation treatment (No or Yes), chemotherapy (No or Yes), tumor size, and the histologic type (LMS NOS, Epithelioid LMS, Bizare LMS, and Myxoid LMS). The primary site was divided into uterus, soft tissue, retroperitoneum, others (eye and orbit, bones and joints, other digestive organs, trachea mediastinum, and other respiratory organs), distant metastasis (DM; No metastasis, oligo metastasis, and multiple metastases). The survival analysis' primary outcome was the overall survival (OS), defined as the time from diagnosis to death due to any cause.

### 2.3 Statistical analysis

The eligible patients were randomly divided into training set (*n* = 2184, 70%) and testing set (*n* = 652, 30%). In this study, patients in the training set were used to establish a nomogram, and patients in the test group were used to verify the nomogram. In this study, *p‐*value <.05 (bilateral) was considered statistically significant. Univariate and multivariate logistic regression models were used to analyze the risk factors of DM in LMS patients. Based on these independent risk factors, a nomogram was established by R software. The nomogram was then evaluated by receiver operating characteristic curve (ROC), calibration curve analysis, and decision curve analysis (DCA). A web‐based nomogram was further prepared based on the nomogram by the “Dynnom” package. The survival time was measured by the Kaplan–Meier analyzes, and the difference between DM and without DM was tested by log‐rank test. Cox proportional hazard regression model was used for univariate and multivariate analysis, and significant variables were obtained. All statistical analyses were performed using R software (http://www.Rproject.org, version 4.0.3).

## 3 RESULTS

### 3.1 Demographic and clinical characteristics

**FIGURE 1:**
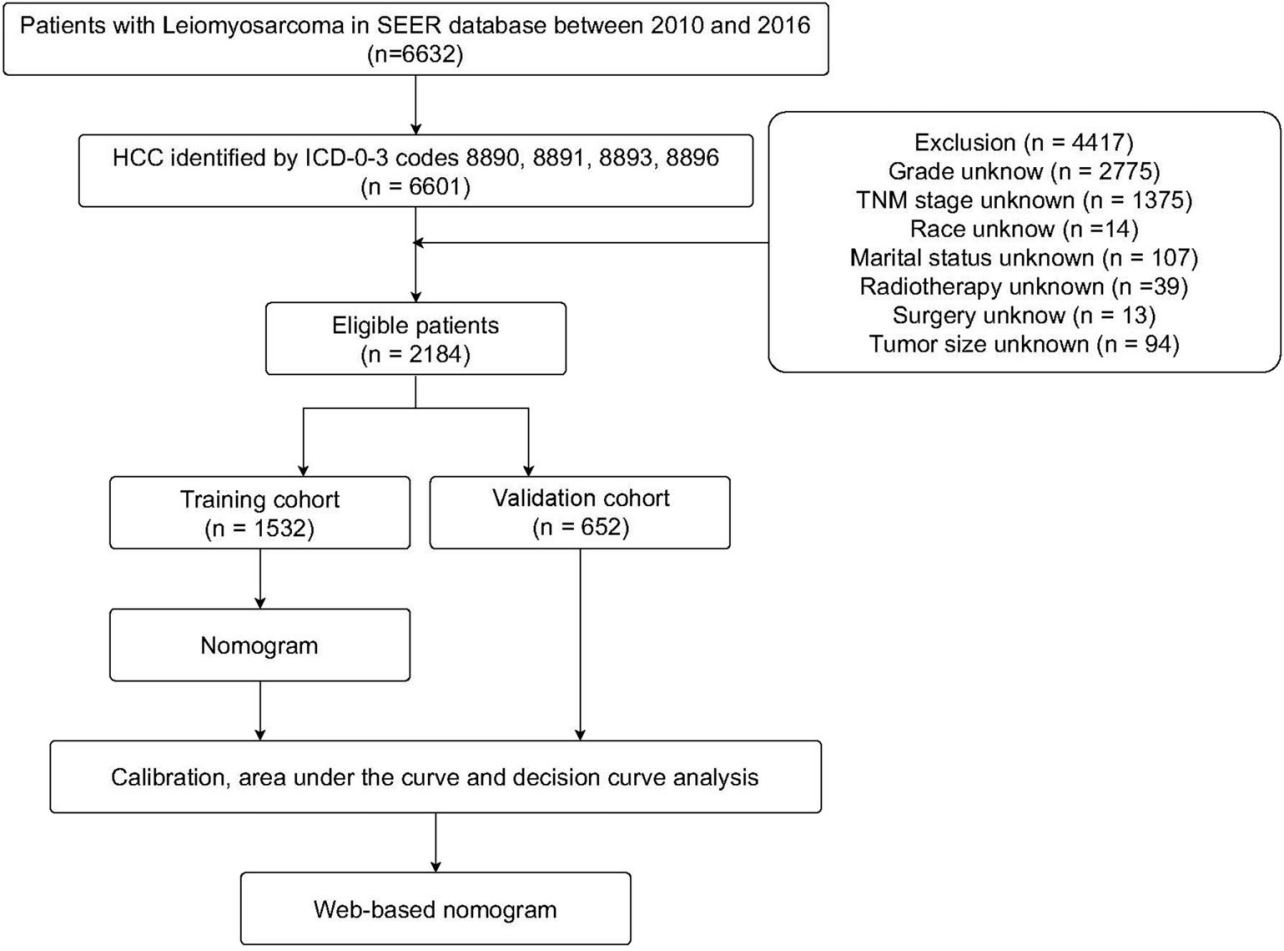
The flow chart of the study design and analysis

A detailed workflow was shown in Figure 1. According to the predetermined criteria, a total of 2184 LMS patients were included. There were 699 males (32.01%) and 1485 females (67.99%). As for race, most of the patients were White (*n* = 1667 [76.33%]), followed by Black (*n* = 324 [14.84%]) and Others (*n* = 193 [8.84%]). The most common site of primary tumor is soft tissue (*n* = 1176 [53.85%]), followed by uterus (*n* = 668 [30.59%]), retroperitoneal (*n* = 300 [13.74%]), and others (*n* = 40 [1.83%]). The most common histological type was LMS NOS (*n* = 2103 [96.29%]), the others (Epithelioid LMS, Bizare LMS, and Myxoid LMS) were 81 cases (3.71%). Differentiation in grades III–IV (*n* = 1434, 65.66%) was the most common among tumor classifications. T1–2 (*n* = 2068, 94.69%) and N0 (*n* = 2098, 6.68%) phases were common. Of all the patients, 1185 (86.31%) had no metastasis, 222 (10.16%) had oligo metastasis and 77 (3.53%) had multiple metastases. Treatment methods selected by LMS patients included surgery (*n* = 1995 [91.35%]), chemotherapy (*n* = 1489 [68.18%]), and radiotherapy (n = 1467 [67.17%]). More details are shown in Table 1.

**TABLE 1:** Demographic and clinical characteristics of patients diagnosed with LMS in SEER database from 2010 to 2016

Subject characteristics	Total cohort		Training cohort		Validation cohort	
	*n*	%	*n*	%	*n*	%
Age						
Median (range, years)	61 (1–101)		61 (2–97)		60 (6–101)	
Sex						
Male	699	32.01	489	31.92	210	32.21
Female	1485	67.99	1043	68.08	442	67.79
Race						
White	1667	76.33	1174	76.63	493	75.61
Black	324	14.84	227	14.82	97	14.88
Others	193	8.84	131	8.55	62	9.51
Marital status						
Married	1746	79.95	1217	79.44	529	81.13
Unmarried	438	20.05	315	20.56	123	18.87
Primary site						
Soft tissue	1176	53.85	835	54.50	341	52.30
Retroperitoneum	300	13.74	204	13.32	96	14.72
Uterus	668	30.59	467	30.48	201	30.83
Others	40	1.83	26	1.70	14	2.15
Histologic type						
LMS NOS	2103	96.29	1483	96.80	620	95.09
Epithelioid	51	2.34	28	1.83	23	3.53
Bizare	2	0.09	1	0.07	1	0.15
Myxoid	28	1.28	20	1.31	8	1.23
Grade						
I–II	750	34.34	534	34.86	216	33.13
III–IV	1434	65.66	998	65.14	436	66.87
T stage						
T1–T2	2068	94.69	1455	94.97	613	94.02
T3–T4	116	5.31	77	5.03	39	5.98
N stage						
N0	2098	96.06	1469	95.89	629	96.47
N1	86	3.94	63	4.11	23	3.53
Number of metastasis						
0	1885	86.31	1309	85.44	576	88.34
1	222	10.16	162	10.57	60	9.20
>1	77	3.53	61	3.98	16	2.45
Tumor size						
Median (range, mm)	85 (1–989)		85 (1–989)		85 (1–989)	
Surgery						
Yes	189	8.65	142	9.27	47	7.21
No	1995	91.35	1390	90.73	605	92.79
Radiotherapy						
Yes	717	32.83	500	32.64	217	33.28
No	1467	67.17	1032	67.36	435	66.72
Chemotherapy						
Yes	695	31.82	478	31.20	217	33.28
No	1489	68.18	1054	68.80	435	66.72
Vital status						
Alive	1258	57.60	877	57.25	381	58.44
Dead	926	42.40	655	42.75	271	41.56

### 3.2 Risk factors for DM development in LMS patients

**FIGURE 2:**
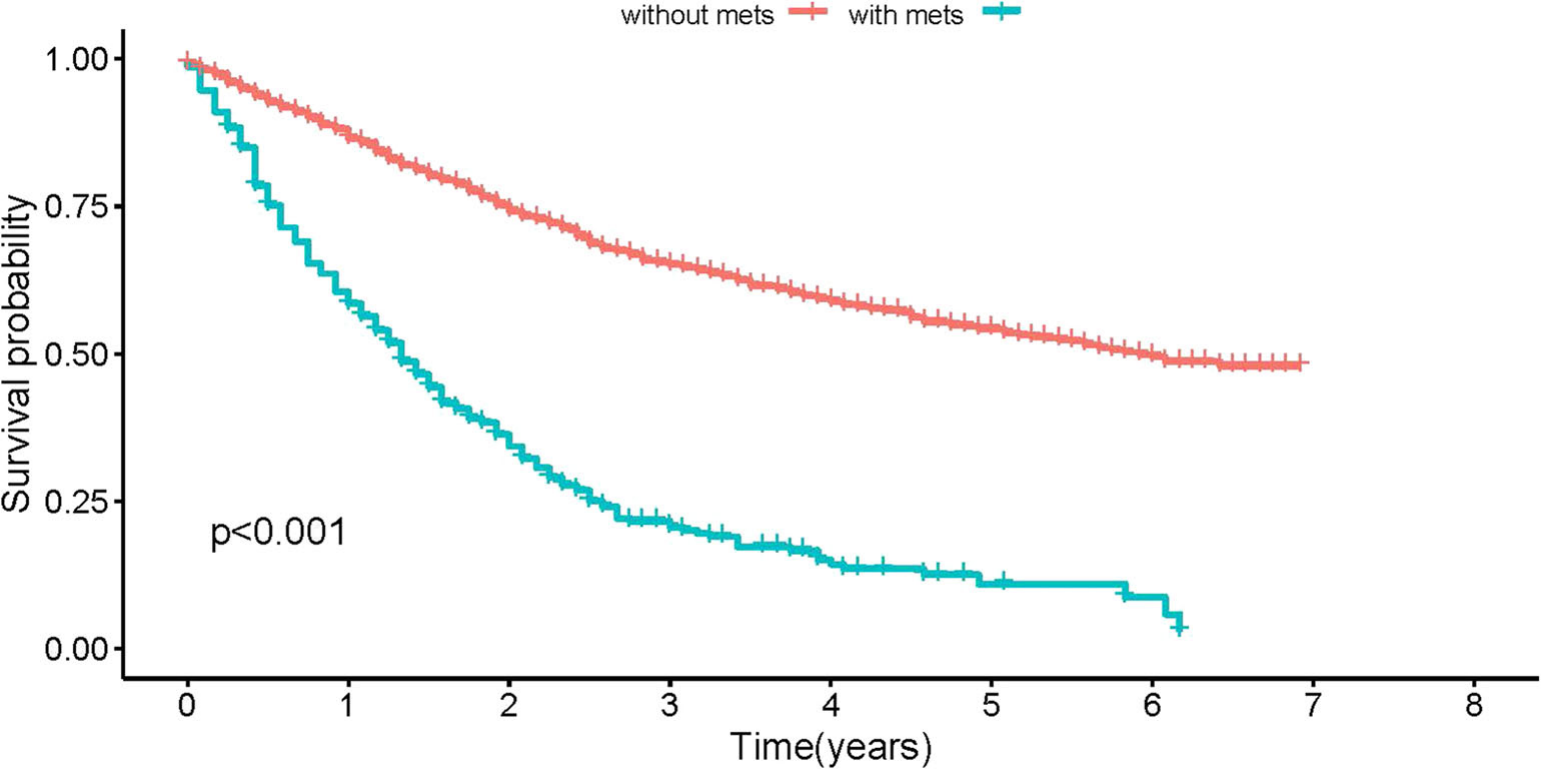
The overall survival for patients with or without distant metastasis

An odds ratio (OR) greater than 1 indicates that the exposure is a risk factor, a OR less than 1 indicates a protective factor, and a value equal to 1 indicates an unrelated factor.^13^ Age, sex, race, grade, T stage, N stage, site, size, and histologic type were related to LMS developing DM in univariate logistics analysis. In multivariate logistics analysis, the Black (OR = 1.445, 95% CI = 1.039–2.008, *p*‐value = .028), grade III–IV (OR = 2.873, 95% CI = 2.030–4.067, *p*‐value < .001), N1 stage (OR = 3.428, 95% CI = 2.125–5.532, *p*‐value < .001), primary site in uterus (OR = 1.754, 95% CI = 1.239–2.483, *p*‐value = .002) and tumor size (OR = 1.002, 95% CI = 1.001–1.004, *p*‐value < .001) were risk factors for DM in LMS patients. More details are shown in Table 2 and Figure 2.

**TABLE 2:** Logistic regression model for analyzing the risk factors for developing distant metastases in patients diagnosed with LMS

	Univariate			Multivariate		
	OR	95%CI	*P*‐value	OR	95%CI	*P*‐value
Age						
Range (years)	0.991	0.983–0.999	.034	0.996	0.986–1.005	.349
Sex						
Male	Reference			Reference		
Female	1.449	1.098–1.912	.009	0.859	0.609–1.211	.386
Race						
White	Reference					
Black	1.627	1.188–2.227	.002	1.445	1.039–2.008	.028
Others	1.291	0.852–1.956	.228	1.234	0.803–1.898	.338
Primary site						
Soft tissue	Reference			Reference		
Retroperitoneum	1.152	0.772–1.717	.489	1.033	0.683–1.562	.879
Uterus	2.312	1.774–3.013	<.001	1.754	1.239–2.483	.002
Others	0.707	0.215–2.327	.568	0.662	0.197–2.229	.506
Histologic type						
LMS NOS	Reference			Reference		
Epithelioid	1.971	1.020–3.809	.044	1.23	0.615–2.460	.559
Bizare	0	0	.999	0	0	.999
Myxoid	0.769	0.231–2.562	.668	0.661	0.191–2.287	.514
Grade						
I–II	Reference			Reference		
III–IV	3.573	2.553–5.001	<.001	2.873	2.030–4.067	<.001
T stage						
T1–2	Reference			Reference		
T3–4	2.333	1.51.‐3.604	<.001	1.001	0.614–1.634	.995
N stage						
N0	Reference			Reference		
N1	4.064	2.576–6.410	<.001	3.428	2.125–5.532	<.001
Size						
Range (mm)	1.003	1.002–1.004	<.001	1.002	1.001–1.001	<.001

### 3.3 Diagnostic nomogram development and validation

**FIGURE 3:**
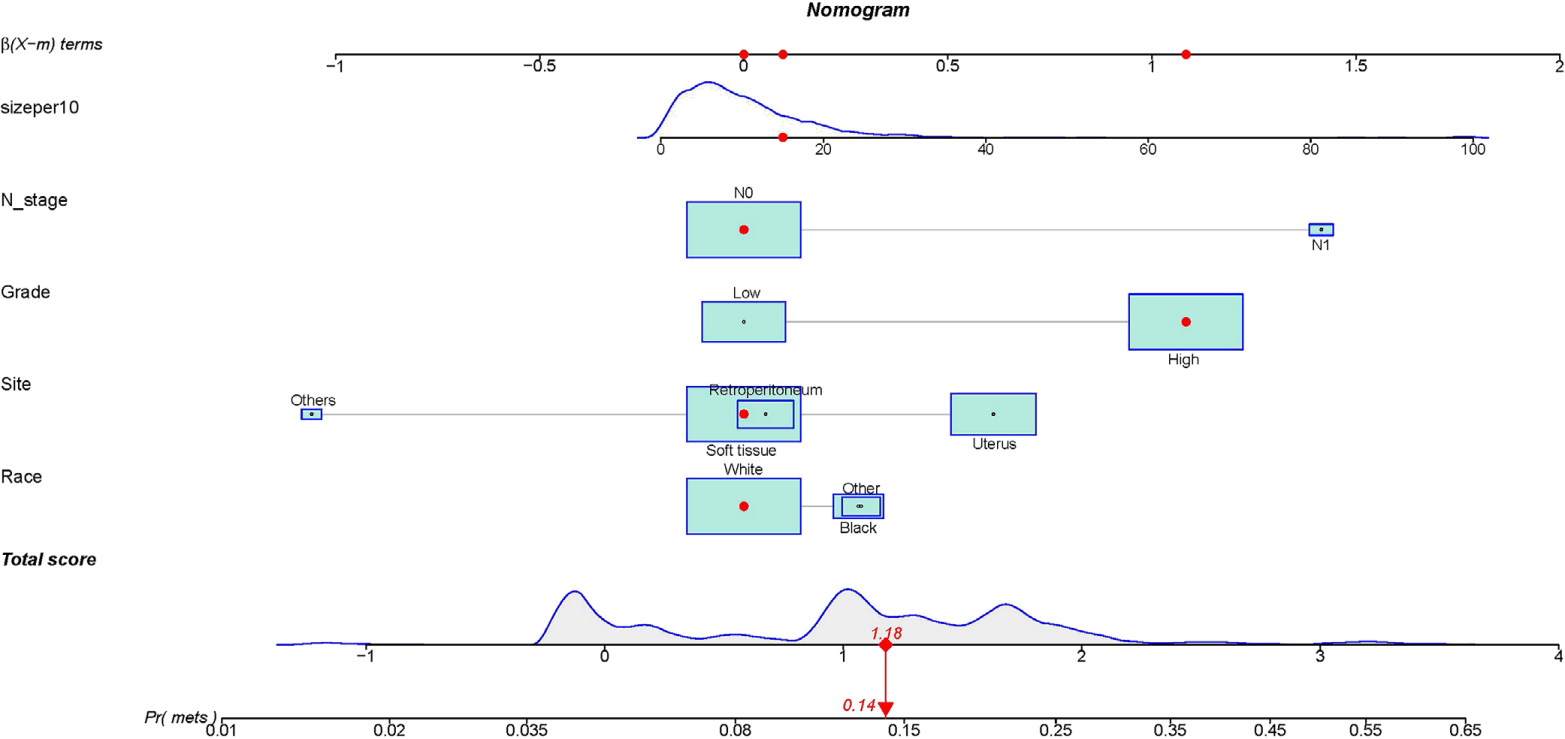
Nomogram to estimate the risk of DM in patients with LMS

**FIGURE 4:**
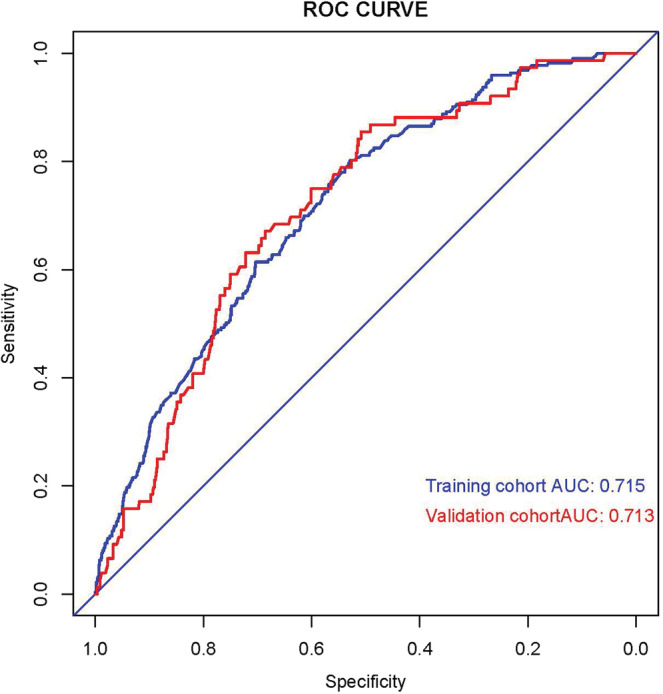
ROC curves of the nomogram for predicting OS in the training cohort (Blue) and the validation cohort (Red)

**FIGURE 5:**
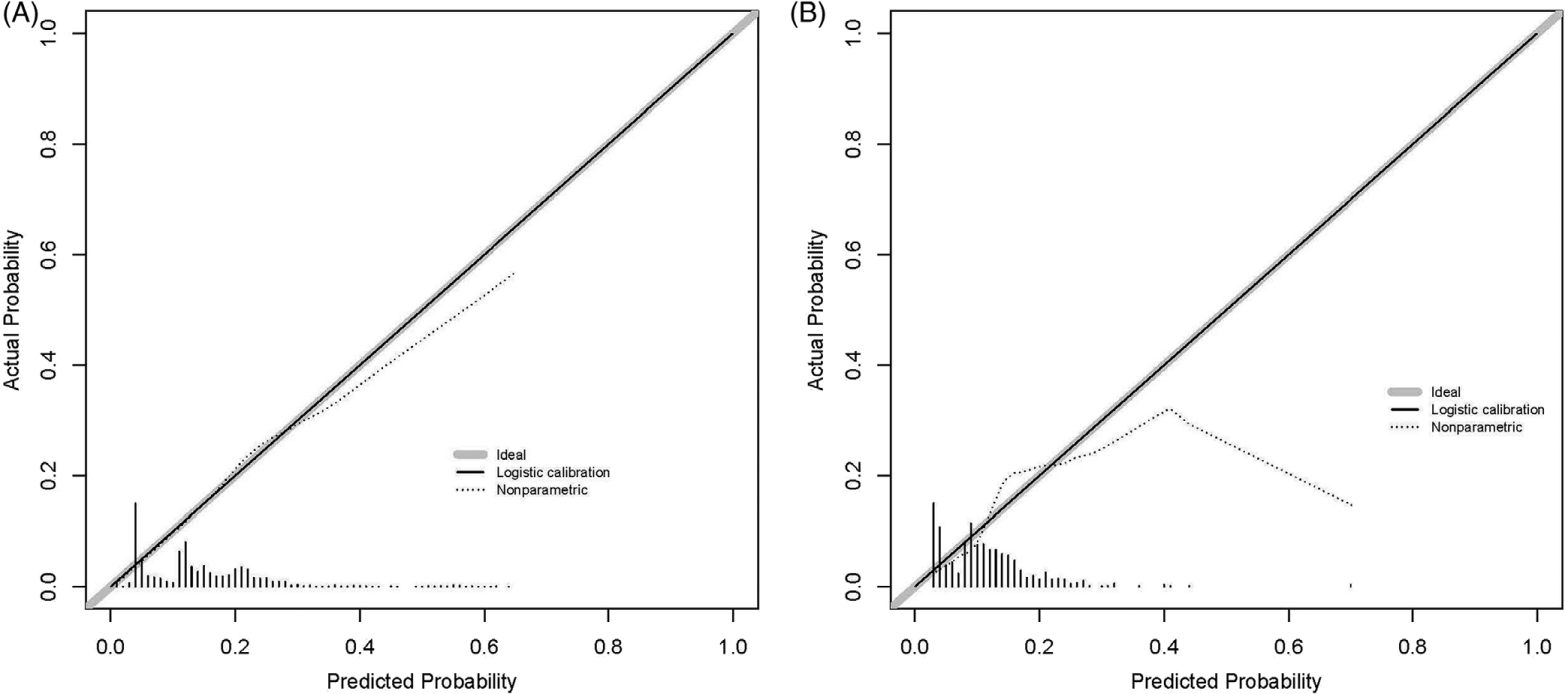
Calibration curves of the nomogram for the risk of LMS with DM in the training cohort (A) and the validation cohort (B), respectively

**FIGURE 6:**
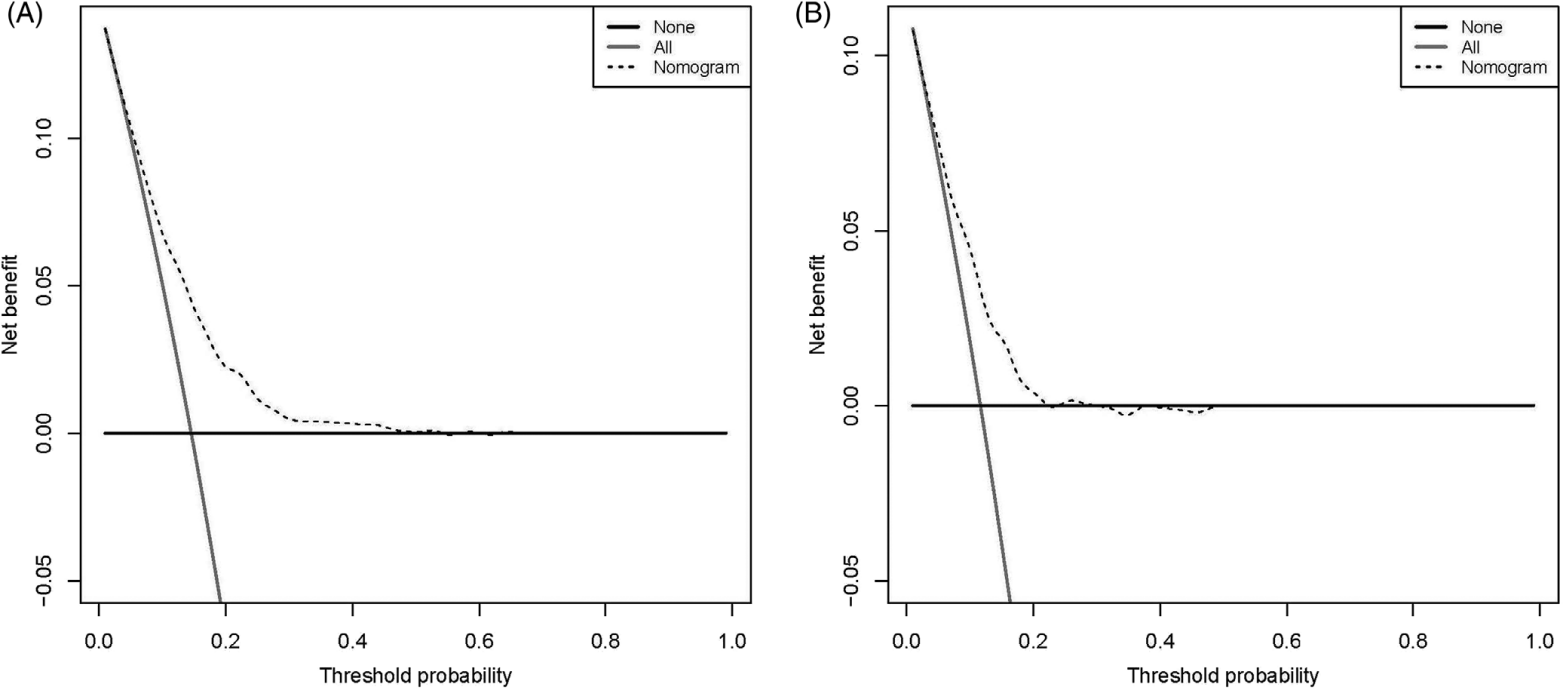
Decision curve analysis of the nomogram for estimating the risk of LMS with DM in the training cohort (A) and validation cohort (B), respectively

We constructed a nomogram according to the logistics regression analysis results, including all risk factors for DM in LMS patients (Figure 3). The area under the curve (AUC) of the nomogram is 0.715 in the training and 0.713 in the validation set (Figure 4). The calibration curve shows a high degree of agreement between the nomogram's predicted results and the desired results in the training set (Chi‐square = 5.236, *p*‐value = .813, Figure 5A) and the validation set (Chi‐square = 7.171, *p*‐value = .619, Figure 5B). Besides, DCA shows that the nomogram can be used as an excellent model to infer the risk of LMS with DM in the training set (Figure 6A) and the validation set (Figure 6B).

### 3.4 The web‐based nomogram

**FIGURE 7:**
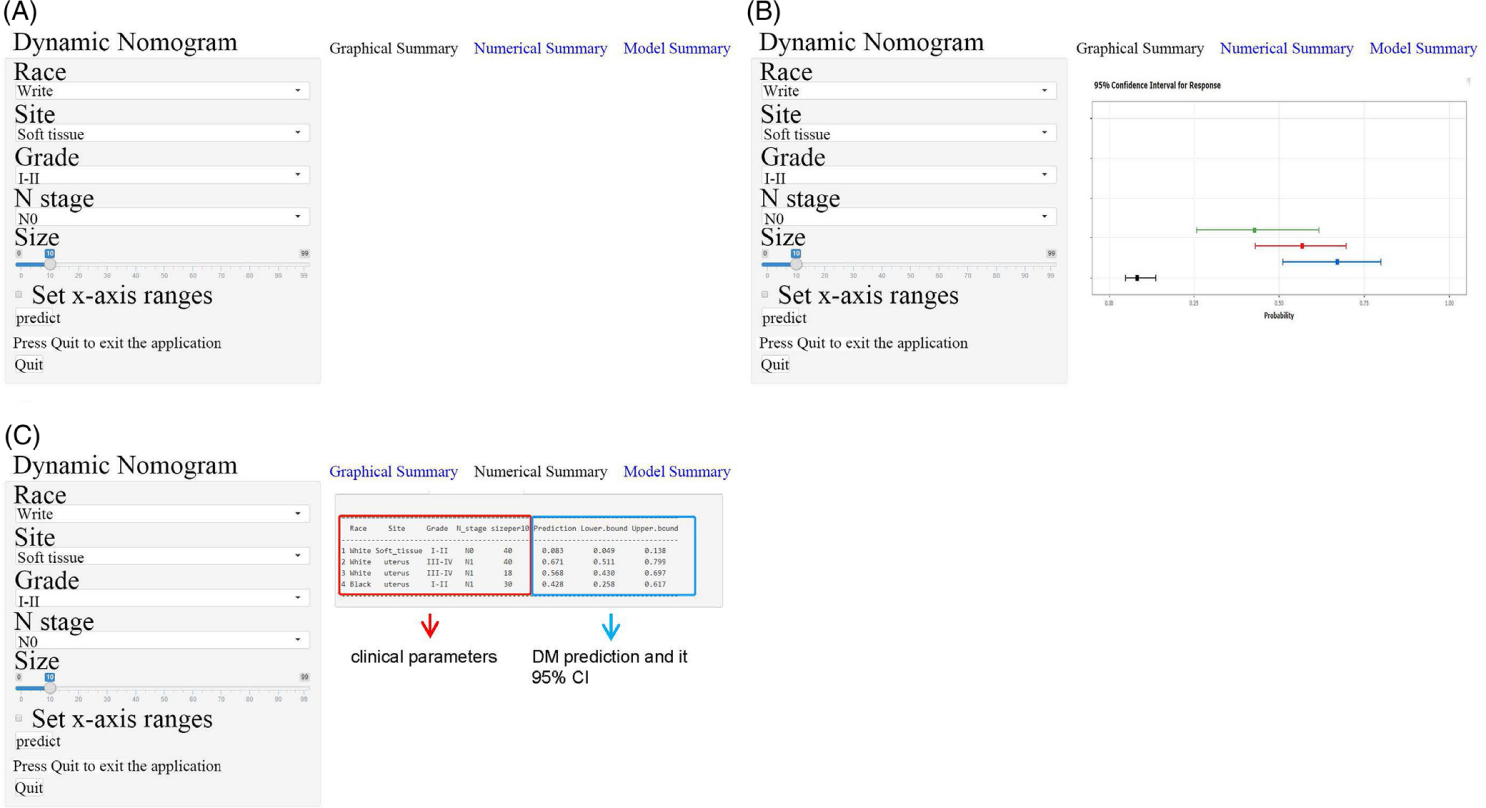
The operation interface of the nomogram on the web page. After entering a patient's Race, Site, Grade, N stage, and Size on https://wenn23.shinyapps.io/riskoflmsdm/, the clinicians can get the LMS patient's corresponding probability of developing DM. (A) Input interface, you can enter a patient's Race, Site, Grade, N stage, and Size in this interface. (B) Graphical summary represents LMS patients' corresponding probability and 95% confidence intervals of developing DM. (C) Numerical summary shows the actual values of probability and 95% confidence intervals

A web version (https://wenn23.shinyapps.io/riskoflmsdm/) was constructed. On the left side of the page are our extrapolated risk factors for DM. According to the patient's condition, clinicians can select the corresponding features in the left interface. Click on the “predict” button, and the right screen shows the prediction of the patient's risk of DM and the specific 95% confidence interval. To help others better understand the operation process of a web‐based nomogram, we randomly enumerate four virtual cases in Figure 7. The four different colored curves in part B represent the risk of DM and the 95%CI for different virtual cases. Part C reflects the specific values.

### 3.5 Survival outcome and prognostic factors for LMS patients with DM

A hazard ratio (HR) less than 1 indicates a protective effect; a HR greater than 1 indicates a detrimental effect. Univariate Cox analysis showed that age, tumor size, number of metastases, histological type, surgery, and chemotherapy were associated with OS. In multivariate Cox analysis, only age (HR = 1.012, 95% CI = 1.001–1.022, *p*‐value = .026), tumor size (mm) (HR = 1.004, 95% CI = 1.002–1.006, *p*‐value < .001), Epithelioid LMS (HR = 2.369, 95% CI = 1.304–4.306, *p*‐value = .005), multiple metastases (HR = 1.48, 95% CI = 1.246–2.195, *p*‐value < .001), surgery performed (HR = 1.895, 95% CI = 1.404–2.558, *p*‐value < .001), and chemotherapy performed (HR = 1.654, 95% CI = 1.246–2.195, *p*‐value < .001) were independent prognostic indicators of OS. More details are listed in Table 3.

**TABLE 3:** Cox proportional hazard regression model for analyzing the prognostic factors for LMS patients with distant metastases

	Univariate			Multivariate		
	HR	95% CI	*P*‐value	HR	95%CI	*P*‐value
Age	1.012	1.002–1.023	.017	1.012	1.001–1.022	.026
Sex						
Male	Reference					
Female	1.165	0.864–1.571	.318			
Race						
White	Reference					
Black	0.997	0.721–1.380	.986			
Others	0.862	0.556–1.336	.057			
Primary site						
Soft tissue	Reference					
Retroperitoneum	0.938	0.617–1.428	.764			
Uterus	1.233	0.934–1.628	.139			
Others	1.344	0.330–5.472	.68			
Histologic type						
LMS NOS	Reference			Reference		
Epithelioid	1.852	1.003–3.318	.038	2.369	1.304–4.306	.005
Myxoid	0.702	0.174–2.829	.619	0.605	0.149–2.466	.483
Grade						
I–II	Reference					
III–IV	1.385	0.938–2.045	.102			
T stage						
T1–T2	Reference			Reference		
T3–T4	2.108	1.401–3.171	<.001	2.895	1.861–4.506	<.001
N stage						
N0	Reference					
N1	1.112	0.743–1.664	.606			
Radiotherapy						
Yes	Reference					
No	1.058	0.788–1.422	.706			
Chemotherapy						
Yes	Reference			Reference		
No	1.36	1.040–1.777	.025	1.654	1.246–2.195	<.001
Number of mets						
1	Reference			Reference		
≥2	1.735	1.298–2.319	<.001	1.48	1.089–2.012	.012
Marital status						
Yes	Reference					
No	0.742	0.546–1.009	.057			
Surgery						
Yes	Reference			Reference		
No	1.813	1.376–2.390	<.001	1.895	1.404–2.558	<.001
Size	1.004	1.002–1.006	<.001	1.004	1.002–1.006	<.001

### 3.6 Survival outcome for patients with DM

For patients without metastases, the 1‐, 2‐, and 3‐year survival rates were 86.5%, 74.2%, and 65.1%, respectively. The median OS was 71 months. However, for metastatic patients, the 1‐, 2‐, and 3‐year survival rates were 58.5%, 34.3%, and 20.6% with a median OS of 16.0 (95% CI: 13.622–18.378) months. The trend of OS for LMS patients with or without initial DM is illustrated in Figure 2.

## 4 DISCUSSION

LMS is an aggressive tumor of soft tissue sarcoma, and about 30% of LMS patients are prone to metastasize to distant organs.^10^, ^14^, ^15^ Existing evidence indicated that the median survival time of LMS patients with lung metastasis is 15 months.^3^, ^16^ Therefore, it is crucial to identify the risk factors of LMS patients developing DM. At the same time, early intervention should be carried out for patients prone to DM to prolong the survival period. However, few studies have explored the risk of DM in LMS patients, and there was no relevant research on the web‐based nomogram. Unlike previous nomograms, visualized web‐based nomograms can accurately predict the risk of DM. The clinician can select the corresponding variable on the left side of the page according to the conditions of different patients to obtain the risk of patients with DM (Figure 7). It is an effective tool for developing personalized follow‐up plans and providing health counseling. Therefore, we first established the web‐based nomogram about the risk of DM in LMS patients based on the SEER database.

Previous studies have shown that grade is considered the most important prognostic factor of LMS and is also a predictive index of DM.^1^, ^17^ In our study, we also found that high‐grade LMS patients were more likely to develop DM. Additionally, it is worth noting that although lymph node (LN) metastasis is rare in LMS patients (*n* = 86, 3.94%), once LN occurs, it indicates that patients have a higher probability for DM (OR = 3.428, 95% CI = 2.125–5.532, *p*‐value < .001). This observation was consistent with previous studies.^18^ A retrospective study has also shown that uterus LMS patients have a worse prognosis than non‐uterine.^19^ In Lamm's opinion, compared with the retroperitoneal and extremity LMS, the uterus LMS is associated with a worse prognosis owing to late detection and negative clinical features.^19^ In contrast to Lamm's prediction, we further classified non‐uterine tissues into soft tissues, retroperitoneum, and others. Our study showed that the prognosis of primary uterine LMS was the worst (OR = 1.754, 95% CI = 1.239–2.483, *p*‐value = .002), and the prognosis of retroperitoneal LMS was similar to that of soft tissue LMS (OR = 1.033, 95% CI = 0.683–1.562, *p*‐value = .879). Because LMS in other sites is rare (*n* = 40, 1.83%), it is not enough to infer clinically significant results. Compared with Lamm's research, we use the web‐based nomogram to visually evaluate the prognosis of LMS patients with different characteristics and predict the risk of DM. Accumulating evidence demonstrated that early surgery and a negative surgical margin greatly reduce the potential of local recurrence and DM.^20^, ^21^, ^22^ Therefore, surgery is considered to be an important factor in the prognosis of patients. In addition, it should be noted that advanced age is associated with tumor metastasis and leads to a poor prognosis. Therefore, we hypothesized that this poor prognosis and higher DM risk were associated with poor physical function in older patients, who often suffer from chronic diseases. The benefit of chemotherapy on the survival of LMS patients is controversial. Accumulating researches have shown that chemotherapy is an important factor in improving the prognosis of patients.^23^, ^24^ However, other research concluded that adjuvant chemotherapy is not associated with significant survival benefits.^25^, ^26^ Our findings support that chemotherapy improves the prognosis of LMS patients with DM. Besides that, LMS patients with multiple metastases were worse than those with oligo metastasis. Multiple metastases are closely related to the imbalance of multiple organ functions and the decline of patients' quality of life.^27^

The present study had some limitations. First of all, the design of this study is a retrospective study, and selection bias is inevitable. Secondly, because the SEER database does not provide the exact surgical method, surgical margin distance, specific methods of chemotherapy and radiotherapy, and the severity of DM, we cannot get the impact of the above dates on LMS patient's survival. The third limitation is that the order of treatment is not considered. Since the data set does not record relapse or progression, we must consider a baseline variable rather than a time‐variant variable. We hypothesized that the exact combination of treatments was determined at the time of diagnosis. This assumption is necessary to integrate treatment information into the model in the absence of precise treatment timing. The fourth limitation is that the study only uses internal verification methods to verify the clinical application value of nomograms and lacks external verification. This shortcoming is also what our research group needs to improve in the next step. Finally, we included only patients diagnosed with LMS from 2010 to 2016, a more extensive time range and larger sample size may help to improve the reliability and persuasiveness of prediction further.

Despite the limitations of this study, the advantages of this study are as follows. First of all, the specific study methods and statistics involved in the nomogram were used to synthesize the baseline characteristics of patients with LMS. Results from this analysis can be used to predict DM in LMS patients. Secondly, in our study, the nomogram showed excellent performance in DM risk assessment, which will enable more accurate personalized clinical decision making and monitoring. Thirdly, to the best of our knowledge, our study is the first to focus on predicting the risk of DM for LMS patients. The results can be used as a basis for personalized treatment. Finally, the web‐based nomogram is performed based on the nomogram, which has a friendlier window than the conventional nomogram and provides a more convenient and intuitive forecast probability.

## 5 CONCLUSIONS

In conclusion, training and validation of the nomogram based on prognostic factors can provide satisfactory predictive efficiency. To encourage widespread clinical use, we developed a web‐based nomogram (https://wenn23.shinyapps.io/riskoflmsdm/). It is an auxiliary graphical tool to evaluate the risks of DM in LMS patients. Advance age, epithelioid histologic type, larger tumor size, multiple metastases, no chemotherapy performed, and no surgery performed associated with worse survival in LMS patients with DM.

## CONFLICT OF INTEREST

The authors declare there is no conflict of interest.

## AUTHOR CONTRIBUTIONS

*Conceptualization, Methodology, Software, Data Curation, Visualization, Formal analysis, and Writing ‐ Original Draft,* Z.L.; *Conceptualization, Methodology, Data Curation, Writing ‐ Original Draft, and Writing ‐ Review & Editing*, J.W.; *Validation, Formal analysis, Data Curation, and Writing ‐ Original Draft,* H.C.; *Data Curation, Validation and Formal analysis,* M.S.; *Data Curation, Validation and Formal analysis,* Y.Z.; *Writing ‐ Review & Editing, Supervision, and Project administration,* Y.J.

## ETHICS STATEMENT

We have obtained permission to access research data files in the SEER program of the National Cancer Institute (reference number 18284‐Nov2019). No ethical review is required because SEER data is publicly available and has been de‐identified.

## Data Availability

The data used in this study are freely accessible and can be obtained via the National Cancer Institute SEER database.
